# Benign Ectopic Thyroid in the Lateral (Level II) Neck Compartment

**DOI:** 10.7759/cureus.22140

**Published:** 2022-02-11

**Authors:** George S Liu, Gerald J Berry, Kaniksha Desai, Uchechukwu C Megwalu

**Affiliations:** 1 Otolaryngology - Head and Neck Surgery, Stanford University School of Medicine, Stanford, USA; 2 Pathology, Stanford University School of Medicine, Stanford, USA; 3 Internal Medicine, Stanford University School of Medicine, Stanford, USA

**Keywords:** clinical endocrinology, ectopic thyroid, general otolaryngology, clinical case report, congenital neck mass

## Abstract

Ectopic thyroid most commonly presents in the midline and is typically associated with the absence of an orthotopic thyroid. Less commonly, ectopic thyroid can present in the lateral neck, typically with a coexisting orthotopic thyroid and abnormal pathology in either the ectopic or orthotopic thyroid tissue. This paper describes a rare case of a benign, ectopic thyroid in the lateral neck (level II) associated with a normal, benign orthotopic thyroid. This report illustrates clinical pearls for the management of this unusual entity.

## Introduction

Ectopic thyroid is uncommon, with an estimated prevalence of 1 in 100,000, and involves the presence of thyroid tissue in an abnormal location, most often in the midline neck near the base of the tongue with an absence of an orthotopic thyroid [1]. Less commonly, ectopic thyroid can occur off midline in the submandibular space (level Ib) or, rarely, in the lateral neck (levels II-IV) [2-6]. We describe a case of benign ectopic thyroid presenting in the right lateral neck (level IIa) with a coexisting benign orthotopic thyroid. Initially, the lesion was suspected to represent cervical metastasis of a thyroid malignancy without a known primary. However, the intraoperative appearance and frozen section analysis of the lateral neck lesion appeared benign, which changed the surgical management and allowed the preservation of the patient’s thyroid gland. This case illustrates clinical pearls for the diagnosis and management of ectopic thyroid in the lateral neck in the rare case that it is encountered. The Stanford Institutional Review Board provided an exemption for this report.

## Case presentation

**Figure 1:**
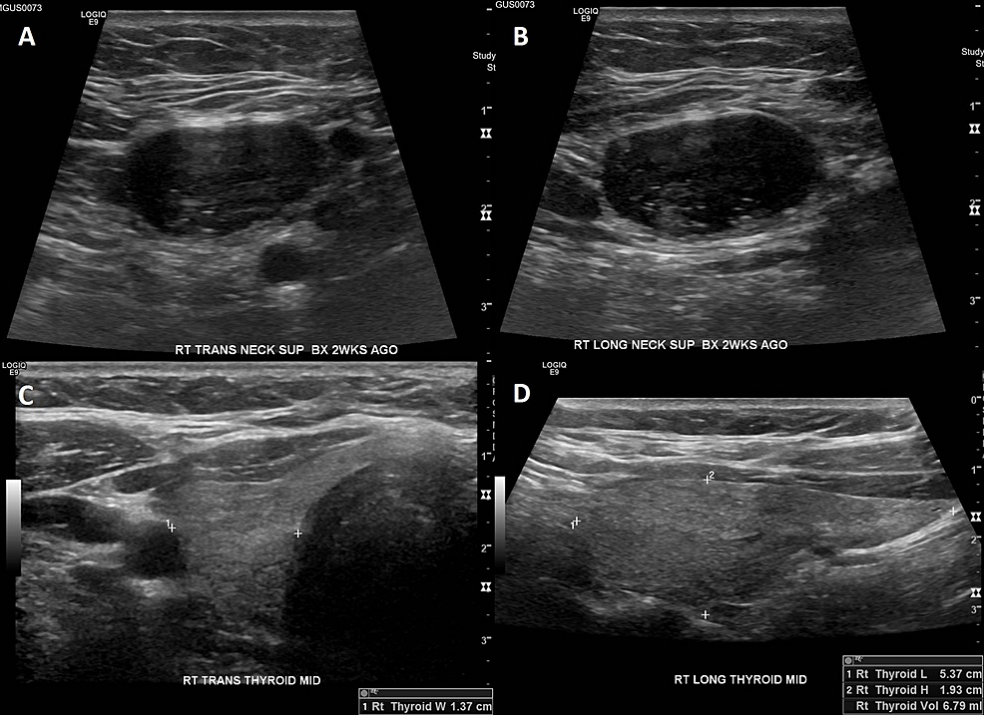
Ultrasound of right neck mass (A) Axial and (B) sagittal ultrasound images of the hypoechoic right neck nodule (level IIa) measuring 2.0 x 1.2 x 2.4 cm. (C) Transverse and (D) sagittal ultrasound images of the right thyroid lobe.

**Figure 2:**
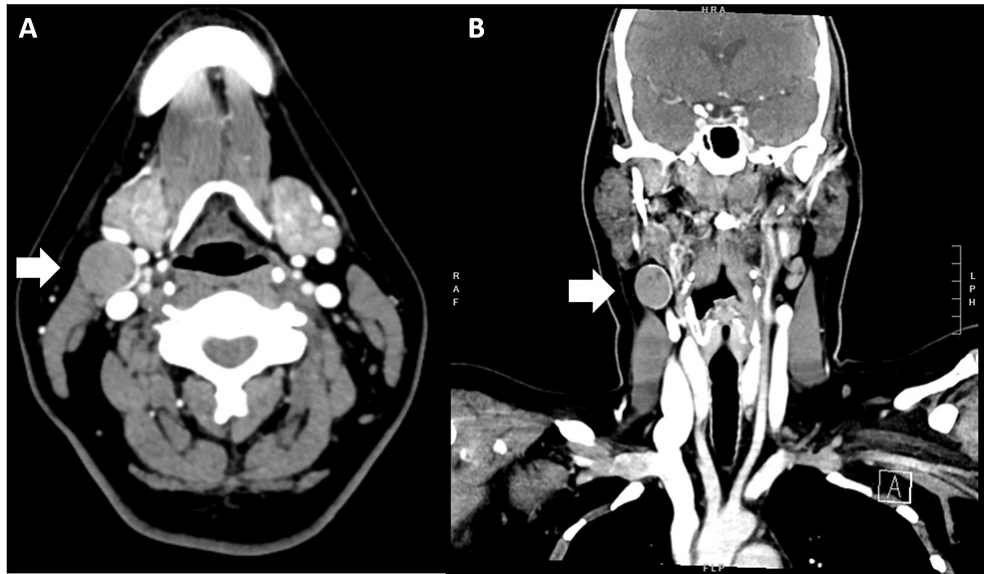
Computerized tomography (CT) scan of the neck with contrast (A) Axial and (B) coronal contrast-enhanced CT images reveal a 1.9 x 2.0 x 2.2 cm soft tissue lesion in the right neck compartment (level IIa) (white arrows) with peripheral enhancement and areas of central low attenuation. There is an associated nodular focus of hyperdensity that could represent enhancement versus calcification. This scan was obtained three months after the fine needle aspiration biopsy of the lesion.

A 49-year-old female presented to our clinic with a painless mass in the right neck that had been asymptomatic and stable in size for 1.5 years. The mass had been first noted incidentally by her dentist. She had no history of radiation exposure and no personal or family history of head and neck cancer. On examination, the mass was soft, non-tender, mobile, and deep to the sternocleidomastoid muscle. Ultrasound (Figure 1), computed tomography (CT) (Figure 2), and magnetic resonance imaging (MRI) demonstrated a 1.5 x 1.8 x 2.1 cm partially cystic lesion in the right neck compartment (level IIa) and a normal, orthotopic thyroid gland with no suspicious nodules. Fine needle aspiration (FNA) of the right neck lesion demonstrated paucicellular cyst fluid with elevated thyroglobulin (>30,000 ng/mL) and no epithelial cells, consistent with a cystic lesion containing thyroid tissue. Because no epithelial cells were recovered, the cytologist was unable to directly visualize thyroid tissue cells in the sample to assess for features concerning for malignancy. Endocrinologic workup revealed her to be euthyroid.

The patient’s case was discussed at our multi-disciplinary thyroid and parathyroid tumor board, which determined that her clinical presentation was most consistent with stage I cT1N1b regionally metastatic papillary thyroid microcarcinoma (PTC) to the right lateral neck without an identified primary lesion on preoperative imaging. It was determined that the primary malignant lesion would likely be found in the orthotopic thyroid gland on final pathology. Therefore, the tumor board recommended performing a total thyroidectomy, central neck dissection, and right lateral neck dissection. The risks, benefits, and alternatives to surgery were discussed with the patient, including the alternative surgical option of a staged approach with initial right neck dissection followed by total thyroidectomy and central neck dissection if the pathology of the neck nodes was positive for thyroid cancer. The patient elected to proceed with upfront total thyroidectomy and neck dissections. She was scheduled for a total thyroidectomy with central and right lateral neck dissections. Intraoperatively, a 3 cm, gray, right neck mass (level II) was noted. The appearance of the mass was smooth and atypical for cervical metastasis of papillary thyroid cancer. Frozen section analysis of the mass demonstrated bland thyroid tissue epithelium without evidence of malignancy. Based on the frozen section results and the atypical appearance of the level II neck mass, the operation was stopped with a plan to stage a completion thyroidectomy and left central neck dissection if final histopathology demonstrated malignancy. The portions of the scheduled surgeries that were performed were right lateral neck dissection, right hemithyroidectomy, and right-central compartment neck dissection.

**Figure 3:**
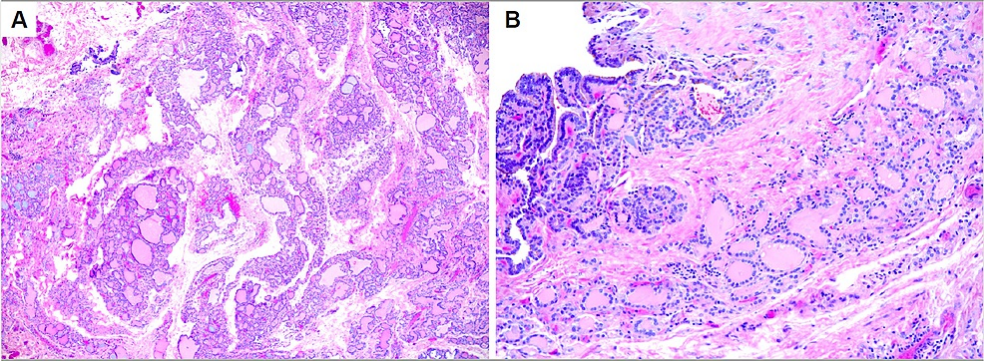
Surgical pathology of right level II neck mass (A) Permanent pathology of right level II neck mass, showing aggregates of benign thyroid tissue arranged in lobules composed of follicles of varying sizes lined by bland follicular epithelium and a circumscribed, unencapsulated nodule composed of bland thyroid epithelium arranged in papillary infoldings (H&E x40). (B) High-power magnification showing a benign cystic lesion composed of papillary infoldings lined by follicular epithelium lacking nuclear features of papillary thyroid microcarcinoma (PTC) (H&E x200). The findings are best classified as a benign thyroid nodule with cystic alterations.

Final histopathology demonstrated the right neck mass to be a benign thyroid nodule with cystic alterations and no evidence of malignancy (Figure 3). The neck dissection specimen contained 45 lymph nodes, all of which were negative for carcinoma. The right thyroid lobe pathology demonstrated benign thyroid parenchyma with multifocal adenomatoid nodules. At the 14-month follow-up, the patient was doing well with no evidence of disease, normal vocal-fold mobility on flexible laryngoscopy exam, and stable subclinical hypothyroidism on low-dose levothyroxine.

## Discussion

To the best of our knowledge, this is the first report in the English-language literature of a benign, ectopic thyroid in the lateral neck (levels II-IV) associated with a normal, benign orthotopic thyroid. Two prior reports describe benign ectopic thyroid in the lateral neck associated with an orthotopic, multinodular thyroid goiter [2,3]. Three prior reports describe ectopic thyroid in the lateral neck associated with PTC involving either the ectopic or orthotopic thyroid gland [4-6]. Table 1 summarizes the findings of these case reports. Based on these case reports and the one presented here, ectopic thyroid tissue in the lateral neck typically presents with the appearance of a cystic nodule and, interestingly, typically coexists with an orthotopic thyroid, in contrast with midline ectopic thyroid, which most commonly lacks an orthotopic thyroid in 70-90% of patients [1,2].

**Table 1:** Reported cases in the literature of ectopic thyroid in the lateral neck (levels II-IV)

Location of ectopic thyroid	# Cases / # Nodules	Characteristic of ectopic nodule	Ectopic thyroid pathology	Orthotopic thyroid pathology	Reference
Right level IV	1 / 1	2 cm cystic	Papillary thyroid carcinoma	Total benign	Sánchez Fuentes et al. 2015 [4]
Left levels II and III	1 / 2	Two 4 cm nodules, calcified and cystic	Benign ectopic thyroid tissue	Left lobe papillary thyroid carcinoma nodule	Choi et al. 2008 [5]
Lateral neck (unspecified)	6 / 6	2/6 palpable, 4/6 on ultrasound (US)	Nodular goiter	Multinodular goiter	Santangelo et al. 2016 [2]
Right levels II and III	1 / 1	2.5 cm cystic mass	Papillary thyroid carcinoma	Benign thyroid	Agosto-Vargas et al. 2017 [6]
Left lateral neck (unspecified)	1 / 3	2.5 cm to 9 cm in size, smooth	Adenomatous goiter	Adenomatous goiter	Nasiru Akanmu et al. 2009 [3]

The embryologic development of ectopic thyroid in the lateral neck remains unclear. One theory is that ectopic thyroid tissue in the lateral neck originates from the ultimobranchial bodies, the neural crest progenitors of parafollicular C cells, in the lateral anlage of the thyroid gland. By contrast, midline ectopic thyroid is thought to arise from endodermal remnants left along the thyroglossal duct tract during the descent of the primordial thyroid gland [1].

Presentations of ectopic thyroid in the lateral neck often mimic the clinical presentation of cervical metastasis of a thyroid malignancy with unknown primary. Detection of thyroid tissue outside of the central neck compartment (level VI) rightly warrants strong suspicion for cervical metastasis of a primary thyroid malignancy until proven otherwise. The rare presentation of an off-midline ectopic thyroid is important to be aware of in cases in which preoperative imaging does not reveal a primary thyroid malignant lesion, and there is strong clinical suspicion for a benign lesion based on intraoperative findings. In such cases, a staged approach can be considered with upfront excisional biopsy and frozen section analysis prior to staged completion of thyroidectomy and central neck dissection. This approach can balance safe and effective oncologic care with reducing the morbidity of potentially avoidable surgery that would predispose the patient to lifelong high-dose thyroid hormone replacement.

## Conclusions

Most cases of ectopic thyroid present in the midline with an absence of an orthotopic thyroid; rarely, ectopic thyroid can present in the lateral neck. This is the first report of a benign, ectopic thyroid in the lateral neck (level II) associated with a normal orthotopic thyroid. The presentation of thyroid tissue in the lateral neck rightly warrants suspicion for cervical metastasis of a primary orthotopic thyroid malignancy. However, the absence of a primary lesion on preoperative imaging and benign intraoperative characteristics, including frozen section analysis, suggests that a staged surgical approach can be considered. In our case, a staged surgical approach confirmed the diagnosis of off-midline ectopic thyroid and spared the patient completion thyroidectomy and the associated morbidity of lifelong dependence on high-dose thyroid hormone supplementation. This report contributes to the limited literature on lateral neck ectopic thyroids and refines their characterization to note their typical coexistence with an orthotopic thyroid, in contrast with midline ectopic thyroids.
